# Effect of Dietary Neutral Detergent Fiber Intake on Improving Fecal Consistency in Gestating Gilts

**DOI:** 10.3390/ani15233455

**Published:** 2025-11-30

**Authors:** Yiwen Ji, Fang Gu, Xuefen Yang, Li Wang, Zongyong Jiang, Fangyuan Shao, Hao Xiao, Kaiguo Gao, Cui Zhu

**Affiliations:** 1 School of Animal Science and Technology, Foshan University, Foshan 528225, China; 2112359059@stu.fosu.edu.cn; 2 State Key Laboratory of Swine and Poultry Breeding Industry, Ministry of Agriculture Key Laboratory of Animal Nutrition and Feed Science in South China, Guangdong Public Laboratory of Animal Breeding and Nutrition, Guangdong Provincial Key Laboratory of Animal Breeding and Nutrition, Guangdong Laboratory for Lingnan Modern Agriculture, Institute of Animal Science, Guangdong Academy of Agricultural Sciences, 1 Dafeng 1st Street, Guangzhou 510640, China; gf13633778817@163.com (F.G.); yangxuefen@gdaas.cn (X.Y.); wangli1@gdaas.cn (L.W.); jiangzy@gdaas.cn (Z.J.); xiaohao@gdaas.cn (H.X.); 3 Key Laboratory of Hunan Province for the Products Quality Regulation of Livestock and Poultry, College of Animal Science and Technology, Hunan Agricultural University, Changsha 410128, China; 4 Cancer Center, Faculty of Health Sciences, University of Macau, Macau SAR, China; fangyuanshao@um.edu.mo

**Keywords:** neutral detergent fiber (NDF), constipation, animal welfare, gestating gilts, reproductive performance

## Abstract

Constipation is a common issue in sows, characterized by dry and hard feces or prolonged absence of defecation, which exerts adverse effects on the overall health and reproductive performance of sows. While dietary neutral detergent fiber (NDF), due to its physicochemical properties, can enhance satiety and promote gastrointestinal health. The present study aims to determine the effect of different dietary NDF intake levels on reducing constipation without impairing the reproductive capacity of gestating sows. A total of 110 primiparous crossbred gilts (Landrace × Yorkshire) were randomly assigned to five groups with different NDF levels of 19.28% (Group A), 21.36% (Group B), 22.08% (Group C), 22.67% (Group D), and 23.43% (Group E), respectively. The daily feed intake was adjusted across groups to ensure a gradual increase in NDF intake from Group A to Group E, while maintaining consistent intake of other nutrients. The results revealed that dietary higher NDF intake (Groups D and E) significantly reduced the rates of constipation and stereotypic behaviors in gilts (*p* < 0.05). The experiment lasted for two parities, starting from day 30 of the first parity gestation to the end of parturition in the second parity. During the second parity, all groups were fed the same diet, and only the reproductive performance of the sows was observed and recorded. Notably, NDF intake had no significant effect on gilts’ first-parity reproductive performance. This study confirms that an appropriate increase in dietary NDF intake can alleviate constipation in gilts. This finding provides critical, targeted references for optimizing nutritional strategies in primiparous gilt management, filling the void in tailored dietary guidelines for this understudied group and offering a practical solution to balance production efficiency and animal stress in commercial pig farming.

## Simple Summary

Gilts often have constipation and show stressed behaviors during pregnancy, which may affect their health and farming costs. This study aimed to find a solution that can address the relevant issues in gilts during pregnancy without affecting their reproductive capacity. We increased the content of neutral detergent fiber (a commonly used fiber indicator in animal feed) in feed by adding ingredients such as alfalfa hay and wheat bran, so as to test the effects of sow feeds with different fiber intakes. We found that increasing the level of neutral detergent fiber in the diet to 22.67% and 23.43% can alleviate constipation, reduce stress-related behaviors, and does not affect reproduction, thereby preserving reproductive efficiency. These findings contribute to our understanding of nutritional strategies that can be employed to optimize sow health and management.

## 1. Introduction

Constipation is a prevalent issue in large-scale pig farms, especially during gestation, as it impairs sow reproductive performance [1,2]. This condition not only leads to severe discomfort and pain in sows but also prolongs the parturition process, potentially leading to abortion in severe cases [3,4]. Beyond immediate impacts, constipation disrupts intestinal microbiota balance, exacerbating oxidative stress and inflammation during parturition [5]. Its etiology is multifactorial, including gastrointestinal metabolic disorders, intestinal nervous system dysfunction, mycotoxin exposure, insufficient exercise, and uterine compression in late gestation [6,7,8].

Key strategies for managing sow constipation include dietary modifications, laxatives, and probiotics. However, long-term use of laxatives (e.g., magnesium potassium sulfate) adversely affects weaned piglet performance [9], while probiotic efficacy varies individually, limiting practical application [10]. In practical production environments, adjusting the dietary fiber intake has emerged as a predominant strategy to relieve constipation in gestating sows [11]. Studies indicate that maintaining a consistent energy intake while increasing dietary fiber enhances the volume of feed, promotes satiety, and stimulates intestinal peristalsis of sows [12,13]. This action aids in mitigating stereotypic behaviors often associated with stress and discomfort stemming from constipation [14,15,16]. Moreover, dietary fiber is instrumental in reshaping the intestinal microbiota, which is beneficial in alleviating gut inflammation, maintaining intestinal integrity, and facilitating regular defecation [17,18].

Neutral detergent fiber (NDF)—encompassing cellulose, hemicellulose, and lignin—more accurately reflects dietary fiber content than crude fiber (which underestimates fiber due to acid-base digestion losses) [19]. Previous study has demonstrated the beneficial effects of increasing dietary NDF intake through different fiber feed ingredients throughout the gestation of multiparous sows [20]. However, the appropriate dietary NDF intake for gestating gilts remains unclear, and the effect of NDF on constipation in gestating gilts has not yet been elucidated.

Therefore, this experiment aims to explore the relationship between different dietary NDF intake and constipation in gilts. We propose the following hypothesis: there exists a range of NDF intake that can alleviate constipation in gilts without adversely affecting their growth performance during gestation. This study is expected to provide key insights for the precise formulation of dietary fiber ratios, thereby reducing the overall stress of gilts while maintaining their production efficiency of gilts.

## 2. Materials and Methods

### 2.1. Ethical Approval

The experimental animals were derived from the Institute of Animal Science, Guangdong Academy of Agriculture Science. The experimental protocol was approved by the Animal Care Committee of the Institute of Animal Science, Guangdong Academy of Agriculture Science, Guangzhou, China, approval number GAASIAS-2016-017.

### 2.2. Animals and Experimental Design

A total of 110 primiparous Landrace × Yorkshire crossbred gilts with consistent genetic background, similar age, and body weight (197.31 ± 1.41 kg) were selected and randomly assigned to 5 groups with 22 replicates per group (1 sow per replicate). The NDF levels of the gestational diets for the first parity across the five groups were 19.28% (Group A), 21.36% (Group B), 22.08% (Group C), 22.67% (Group D), and 23.43% (Group E), respectively. However, the diets were consistent across all groups during the subsequent lactation period, non-gestating period, and second parity gestation. Nutritional requirements were calculated based on the sow production performance provided by the pig farm according to NRC (2012). Feed restriction was imposed during gestation while ensuring their nutritional requirements were met. The experiment lasted for two parities, starting from day 30 of the first parity gestation to the end of parturition in the second parity. During the second parity, the diets were identical across all groups, and only the reproductive performance of the sows was observed and recorded. All sows were housed in gestation crates (2.8 m in length and 0.6 m in width) throughout the experiment. Feeding and management practices followed the routine production protocols of Tongzheng Pig Farm, affiliated with Guangxi Agricultural Reclamation Jinguang Farm Co., Ltd. (Guangxi, China). The feeding amount is shown in Table 1, and the gilts were fed twice daily at 08:00 and 15:00 Beijing Time. Regular ventilation was maintained, and the indoor temperature was around 25 degrees. During the experiment, sows that aborted or developed other diseases affecting the experimental results were promptly culled.

In this experiment, the nutrient requirements of sows were calculated based on dietary net energy level and the reproductive performance of sows at the experimental farm (backfat thickness at mating, 18.50 mm; body weight at mating, 185 kg; gestation length, 114 days; litter size, 12 piglets; average birth weight of piglets, 1.27 kg; body weight gain during gestation, 45 kg, etc.), with reference to the NRC (2012) nutrient requirement model for swine. The formulations and nutrient levels of the corn-soybean meal-based diets are shown in Table 2.

The net energy levels of the experimental groups were sequentially decreased by 0.42 MJ/kg based on the recommended net energy level in NRC (2012) (10.47 MJ/kg). The levels of crude protein, standardized ileal digestible (SID) amino acids, calcium, and phosphorus varied with the net energy level of the diet. The daily feed intake and NDF intake are shown in Table 1, ensuring that the daily nutrient intake levels of sows in all groups were consistent and met the nutrient requirements for gestating sows specified in NRC (2012).

### 2.3. Sample Collection

#### 2.3.1. Blood Sample Collection

On day 60, day 90 of the first parity gestation period, and at 8:00 a.m. on the day of farrowing, 10 fasting sows were randomly selected and fixed from each group for ear vein blood collection. After disinfecting the ear vein sampling site with alcohol cotton balls, 20 mL of blood was collected into heparin sodium-containing blood collection tubes, allowed to stand at room temperature for 15 min, and then centrifuged at 1500 *g*/min at 4 °C for 20 min to prepare plasma. The plasma was then quickly aliquoted into 1.5 mL EP tubes and stored in a −80 °C refrigerator for subsequent determinations.

#### 2.3.2. Fecal Sample Collection and Feces Consistency Scoring

On day 90 of gestation, 20 g of fresh fecal samples were collected from 6 random gestating gilts each group using sterile cotton swabs. Fecal samples were then placed into sterile centrifuge tubes, labelled, and stored in a −80 °C refrigerator for fecal microbiota analysis.

**Figure 1:**
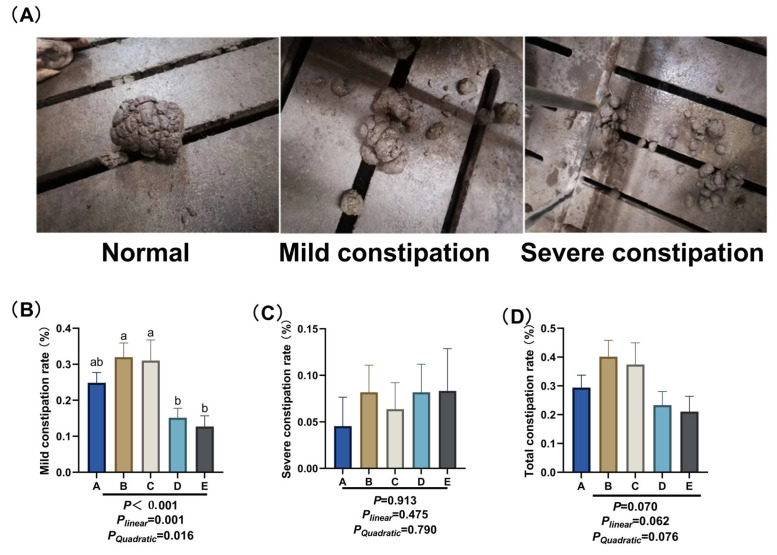
Effect of different dietary NDF intake during pregnancy on constipation in gestating gilts. (**A**) Constipation scoring criteria. Normal: Well-formed feces, soft and bulky, in columnar or banana-like shape; Mild constipation: Slightly hard feces, sized like table tennis balls, with a large main body accompanied by a small amount of small fecal pellets; Severe constipation: Dry and hard feces, small in particle size, mainly composed of small fecal pellets. (**B**) Mild constipation rates. (**C**) Severe constipation rates. (**D**) Total constipation rates. A: NDF levels = 19.28%; B: NDF levels = 21.36%; C: NDF levels = 22.08%; D: NDF levels = 22.67%; E: NDF levels = 23.43%. Differences in the superscript letters within different columns indicate that the difference is significant (*p* < 0.05).

Daily scoring and recording of gilts’ fecal status were initiated from day 90 to day 114 of gestation. Constipation was indirectly assessed based on fecal consistency using the scoring criteria referenced from Tan et al. [17] (Figure 1A). Normal: Well-formed feces, soft and bulky, in columnar or banana-like shape; Mild constipation: Slightly hard feces, sized like table tennis balls, with a large main body accompanied by a small amount of small fecal pellets; Severe constipation: Dry and hard feces, small in particle size, mainly composed of small fecal pellets. The formula for calculating the constipation rate (%) is as follows: (Number of constipated sows during the trial period × Number of constipated days)/(Total number of sows in the group × Number of experimental days) × 100%.

### 2.4. Data Collection and Determinations

#### 2.4.1. Backfat Thickness

The P2 point backfat thickness of sows was measured using an ultrasonic backfat meter (Glawei, China) on day 30, 60, 90 of the first parity gestation and on the day of farrowing, respectively. The specific procedures were as follows: After the sows stood naturally, the location was identified with fingers at 6.5 cm from the dorsal midline at the last rib of the sows. Then, scissors were used to remove the hair on the body surface, and coupling gel was evenly applied. The probe of the backfat meter was placed perpendicular to the sow’s body surface for measurement. Each measurement was repeated three times, and the average value was taken as the backfat thickness at the P2 point.

#### 2.4.2. Body Weight and Feed Intake of Gilts

The body weight of gilts was measured using a floor scale on day 30, day 60, day 114 of the first parity gestation, and on day 21 of lactation, respectively. The daily feed allowance and residual feed weight of gilts were recorded during gestation to calculate daily feed intake. The formula was as follows: daily feed intake = daily feed allowance − daily residual feed (including feed wastage).

#### 2.4.3. Stereotypic Behavior of Gilts

The proportion of gilts exhibiting vacuous chewing was recorded at day 60 and day 90 of gestation, and the number of gilts with vacuous chewing was counted using the instantaneous scan sampling method. Counts were performed in the morning and afternoon, 3 h after feeding, for 7 consecutive days. The criteria for judging gilts with vacuous chewing behavior were sows showing chewing movements, or gilts without chewing movements but with white foam on the edges of the mouth.

#### 2.4.4. Reproductive Performance

At farrowing, reproductive performance such as total number born per litter, number of healthy piglets per litter, number of weak piglets, number of stillborn piglets, number of mummified fetuses, total birth weight per litter, average birth weight per piglet, placenta weight, and farrowing duration were recorded. Among them, newborn piglets with a body weight of over 1000 g, ruddy skin, and strong vitality were identified as healthy piglets. After weaning, the weaning-to-estrus interval was recorded, and the subsequent natural estrus rate and conception rate of the next parity were calculated.

#### 2.4.5. Determination of Plasma Indicators

The contents of total protein (TP), albumin (ALB), urea (UREA), glucose (GLU), triglycerides (TG), cholesterol (CHO), low-density lipoprotein (LDL), high-density lipoprotein (HDL), creatinine (CRE), alanine aminotransferase (ALT), aspartate aminotransferase (AST), and alkaline phosphatase (ALP) in sow plasma were determined using an automatic biochemical analyzer (Selectra Pro XL, VitalScientific, Spankeren, Gelderland, The Netherlands).

The contents of non-esterified fatty acids (NEFA), malondialdehyde (MDA), total superoxide dismutase (T-SOD), catalase (CAT), glutathione peroxidase (GSH-Px), and total antioxidant capacity (T-AOC) in the plasma of gilts were measured using kits from Nanjing Jiancheng Bioengineering Institute (Nanjing, China).

The contents of immunoglobulin A (IgA), immunoglobulin G (IgG), immunoglobulin M (IgM), progesterone (PROG), estradiol (E2), insulin (INS), epinephrine (EPI), ghrelin, and cortisol in the plasma of gilts at 90 days of gestation were determined by enzyme-linked immunosorbent assay (ELISA). The determination methods and calculations were performed following the instructions of the kits from Jiangsu Enzyme-linked Immunology Industry Co., Ltd. (Yancheng, China).

#### 2.4.6. Microbial Community Analysis

Fecal samples were collected from 6 randomly selected sows in each of the five experimental groups, with a total of 30 samples. Total microbial DNA in fecal samples was extracted following the instructions of the E.Z.N.A.^®^ Soil DNA Kit (Omega Bio-tek, Norcross, GA, USA). PCR amplification was performed using primers targeting the V3-V4 region of the bacterial 16S rRNA gene. The sequences of the primers by PCR were as follows: forward primer 5′-CCTAYGGGRBGCASCAG-3′ and reverse primer 5′-GGACTACNNGGGTATCTAAT-3′. All PCR mixtures contained 15 µL Phusion^®^ High-Fidelity PCR Master Mix (New England Biolabs), 0.2 μM primers, and 10 ng genomic DNA template. The PCR program was as follows: initial denaturation at 98 °C for 1 min, followed by 30 cycles of denaturation at 98 °C (10 s), annealing at 50 °C (30 s), and extension at 72 °C (30 s), with a final extension at 72 °C for 5 min. PCR products were subjected to 2% agarose gel electrophoresis, then purified using the AxyPrep DNA Gel Extraction Kit (Axygen Biosciences, Union City, CA, USA) according to the manufacturer’s instructions to recover target products. Library construction was performed, and qualified libraries were sequenced on the NovaSeq 6000 platform with PE250 mode by Novogene Technology Co., Ltd. (Tianjin, China). After assembly, high-quality quality control, and chimera removal of raw data, valid data were obtained. All sequences in the valid data were classified into operational taxonomic units (OTUs) based on different similarity levels. Chimeric sequences were identified and removed using UCHIME v4.1 software, and sequences with 97% similarity were clustered into OTUs using UPARSE v11.0.667 software. Taxonomic analysis of OTU representative sequences was conducted using the RDP classifier Bayesian algorithm, with reference to the Silva138.1 database and a confidence threshold of 0.7. Finally, species information of each OTU at different taxonomic levels was obtained, and the microbial community composition of each sample at each taxonomic level was statistically summarized. Alpha diversity of fecal samples was calculated using 2023.2 release of QIIME2. Beta diversity, reflecting differences in microbial community composition among samples, was compared using Weighted UniFrac principal coordinate analysis (PCoA).

### 2.5. Statistical Analysis

All data are presented as mean ± standard error of the mean (SEM). Prior to intergroup difference analysis, the normality of the data was assessed using the Shapiro–Wilk test, and homogeneity of variance was verified via the Levene test. For variables with a normal distribution and homogeneous variance, one-way ANOVA followed by the LSD test was employed. For non-normally distributed data or data with heterogeneous variance, the Kruskal–Wallis test (a non-parametric alternative to ANOVA) with false discovery rate (FDR) multiple corrections was used. Each gestation stall was considered as one replicate for sow performance data. Statistical analysis was performed using SPSS 25.0 software. One-way analysis of variance (ANOVA) followed by Tukey’s multiple comparison test was used for comparisons among experimental groups. The linear and quadratic effects of dietary NDF levels on gilts and piglet performance were analyzed. Differences were considered significant at *p* < 0.05, and a trend toward significance at 0.05 ≤ *p* < 0.10.

## 3. Results

### 3.1. Constipation Rate

As shown in Figure 1A, after day 90 of gestation, the mild constipation rate of sows decreased significantly with the increase in NDF intake (*p* ≤ 0.01). Specifically, the mild constipation rates of sows in group D and group E were significantly lower than those in group B and group C (*p* < 0.05). As shown in Figure 1B, changing dietary NDF intake had no significant effect on the severe constipation rate. As shown in Figure 1C, with the increse in dietary NDF intake, the total constipation rate tended to decrease (*P_Linear_* ≤ 0.01).

### 3.2. Gilt and Litter Performance

The effects of different dietary NDF intake during gestation on backfat thickness and body weight in primiparous gilts are presented in Table 3. Dietary NDF intake during gestation had no significant effect (*p* > 0.05) on sow backfat thickness at d 30, 60, and 90 of gestation, at farrowing, or at weaning. Similarly, backfat loss, feed intake during lactation, as well as the body weight of sows at d 30 and 60 of gestation, at farrowing, and at weaning were not significantly affected (*p* > 0.05) by gestation dietary NDF intake. However, there was a linear trend (*P_Linear_* = 0.070) toward reducing body weight loss during lactation with decreasing dietary NDF intake during gestation.

The effects of different dietary NDF intake during gestation on reproductive performance over two consecutive parities are presented in Table 4. Dietary NDF intake during gestation had no significant effects (*p* > 0.05) on total number of piglets born, number of live piglets, number of stillbirths, number of mummified fetuses, litter birth weight, placental weight, or duration of parturition. However, a linear trend toward an increased number of low-birth-weight piglets was observed with decreasing dietary NDF intake during the first parity (*P_Linear_* = 0.057). In addition, there was a tendency for dietary NDF intake during gestation to affect the number of mummified fetuses in the first parity (*p* = 0.062), with a significant quadratic effect detected (*P_quadratic_* < 0.01). As shown in Table 4, dietary NDF intake during gestation did not significantly affect piglet body weight on d 0 and d 21, or average daily gain in the first parity (*p* > 0.05).

**Figure 2:**
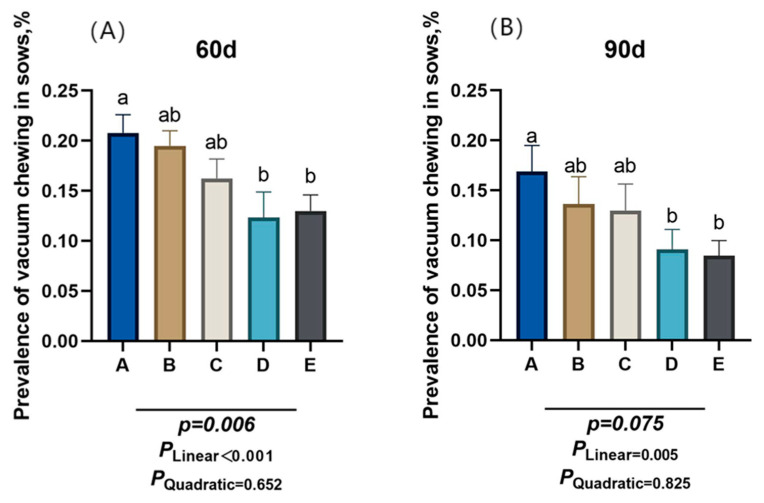
Effect of different dietary neutral detergent fiber intake on the prevalence of vacuum chewing in gestating gilts. (**A**) Day 60 of gestation. (**B**) Day 90 of gestation. A: NDF levels = 19.28%; B: NDF levels = 21.36%; C: NDF levels = 22.08%; D: NDF levels = 22.67%; E: NDF levels = 23.43%. Differences in the superscript letters within different columns indicate that the difference is significant (*p* < 0.05).

As shown in Figure 2, the proportion of sows engaging in oral-nasal chewing behavior decreased linearly (*P_Linear_* < 0.05) at gestation days 60 and 90 as dietary NDF intake increased. Compared to group A, group D and group E had a significantly lower proportion of gilts exhibiting oral-nasal chewing behavior at d 60 of gestation (*p* < 0.05).

### 3.3. Blood Biochemical Parameters

As shown in Table 5, on day 60 of gestation, as the dietary NDF intake decreased, the plasma alkaline phosphatase (ALP) concentration showed a quadratic trend of initial increase followed by a decrease (*P_Quadratic_* = 0.050). The plasma cholesterol (CHO) concentration presented a quadratic decrease followed by an increase (*P_Quadratic_* < 0.01), while the plasma triglyceride (TG) concentration increased linearly (*P_Linear_* < 0.05). Compared with group E, group D showed a significantly increased ALP concentration (*p* < 0.05). Compared with group C, group E showed a significantly higher CHO concentration (*p* < 0.05).

On day 90 of gestation, the plasma total protein (TP) concentration showed a linear increasing trend as NDF intake decreased (*P_Linear_* = 0.053), while the aspartate aminotransferase (AST) concentration exhibited a quadratic trend of increase followed by decrease (*P_Quadratic_* = 0.064). On the day of farrowing, the plasma albumin (ALB) concentration showed a quadratic decrease followed by increase trend (*P_Quadratic_* < 0.05) as dietary NDF intake decreased.

### 3.4. Plasma Antioxidant Capacity of Gilts

As shown in Table 6, on day 60 of gestation, the T-AOC in plasma showed a quadratic increase followed by decrease with declining dietary NDF intake (*P_Quadratic_* < 0.01). Compared with group E, both group D and group B had significantly increased T-AOC levels (*p* < 0.05). The plasma MDA levels were decreased linearly (*P_Linear_* < 0.01) with the increase in dietary NDF intake, with significantly lower MDA levels in group E compared to group B (*p* < 0.05). Moreover, plasma GSH-Px activity were significantly higher in group B than in group E and group D (*p* < 0.05), while catalase (CAT) activity was significantly lower in group D compared to group E (*p* < 0.05).

On day 90 of gestation, plasma T-AOC level continued to increase linearly (*P_Linear_* < 0.01), while plasma CAT activity showed a quadratic change by dietary NDF intake (*P_Quadratic_* < 0.05). Compared with group E, group A had significantly lower T-AOC levels (*p* < 0.05). Similarly, CAT levels in group A were significantly lower than those in E and group B (*p* < 0.05).

At farrowing, plasma T-AOC and GSH-Px activity were not significantly affected (*p* > 0.05) by dietary NDF intake. Compared with group A, group B and E had significantly lower plasma T-SOD levels (*p* < 0.05). Similarly, plasma MDA levels in group A were significantly lower than those in E and group B (*p* < 0.05). The plasma CAT levels of gilts were highly significant lower in group A and group E than in group B and group C (*p* < 0.01).

### 3.5. Plasma Hormone Levels in Gilts

As shown in Table 7, on day 90 of gestation, both plasma progesterone and adrenaline concentrations decreased linearly in gilts as dietary NDF intake decreased (*P_Linear_* < 0.01). Compared with group E, group A exhibited significantly higher plasma levels of progesterone and adrenaline in gilts (*p* < 0.05). Moreover, plasma concentrations of immunoglobulin A (IgA), immunoglobulin M (IgM), and immunoglobulin G (IgG) in gilts on day 90 of gestation decreased linearly with decreasing dietary NDF intake (*P_Linear_* < 0.01). Compared with group E, plasma IgA concentrations in gilts were significantly higher in group C and group A (*p* < 0.05). Moreover, plasma IgM concentrations in gilts were significantly higher in group D and group A (*p* < 0.05), while plasma IgG concentrations in gilts were significantly higher in group A (*p* < 0.05).

### 3.6. Fecal Microbiota of Gilts

**Figure 3:**
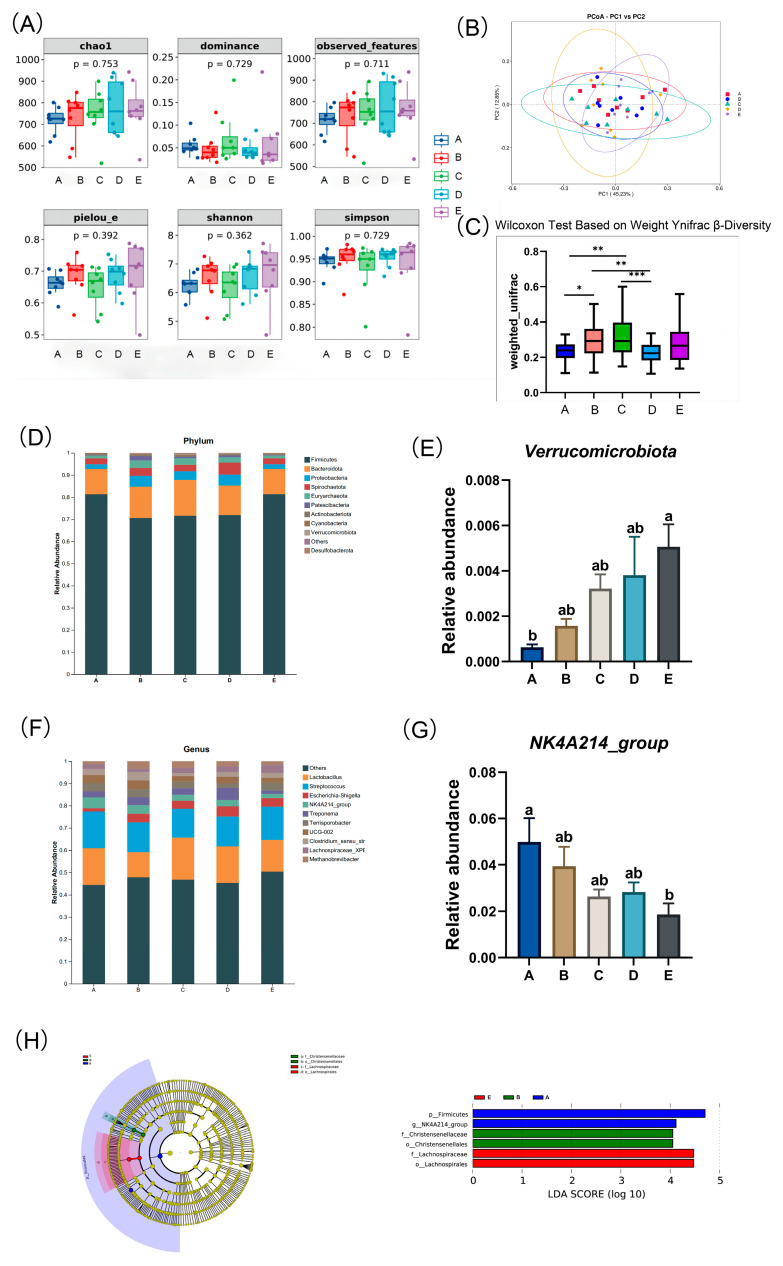
Effect of dietary different neutral detergent fiber intake during pregnancy on fecal microbiota in gilts. (**A**) Alpha diversity. (**B**) Principal Component Analysis. (**C**) Beta diversity. Relative abundance of gut microbiota at phylum (**D**) and genus (**F**) levels. Relative abundance of *Verrucomicrobia* (**E**) and *NK4A214_group* (**G**). (**H**) Linear discriminant analysis Effect Size. A: NDF levels = 19.28%; B: NDF levels = 21.36%; C: NDF levels = 22.08%; D: NDF levels = 22.67%; E: NDF levels = 23.43%. Values are presented as means ± SEM, *n* = 6. Differences were assessed using Tukey’s test for multiple comparisons and denoted as follows: * means a statistical tendency (0.05 < *p* ≤ 0.100), ** means a significant difference (*p* < 0.05), and *** means a highly significant difference (*p* < 0.01). ^a,b^ Values within a row with different superscripts differ significantly at *p* < 0.05.

As shown in Figure 3A, dietary NDF intake had no significant effect on fecal microbial alpha diversity indices, including Chao1, Simpson, Dominance, observed_features, Pielou_e, and Shannon indices in primiparous gestating gilts (*p* > 0.05).

As shown in Figure 3B, the principal coordinates analysis (PCoA) plot based on weighted UniFrac distances depicts the distribution of fecal microbial community structures. Beta diversity indices (Figure 3C), calculated using UniFrac distances, were analyzed through the Wilcoxon test. The results indicated that the beta diversity indices in the C and B groups were significantly higher compared to those in the D and A groups (*p* < 0.05).

As shown in Figure 3D, the relative abundances of the top 10 bacterial taxa at both the phylum and genus levels are presented across different dietary NDF intake during gestation in gilts. The predominant phyla identified were Firmicutes, Bacteroidota, and Proteobacteria, collectively accounting for 89.63% to 94.85% of the total microbial composition across all groups. The remaining phyla, in descending order of proportion, are Spirochaetes, Euryarchaeota, Patescibacteria, Actinobacteria, Cyanobacteria, Verrucomicrobia, Desulfobacteria, and others. As shown in Figure 3E, at the phylum level, the relative abundance of Verrucomicrobia was increased significantly with increasing dietary NDF intake (*p* < 0.05). At the genus level (Figure 3F), Lactobacillus and Streptococcus were dominant. The remaining genera, in descending order of proportion, are *Escherichia-Shigella*, *NK4A214_group*, *Treponema*, *Terrisporobacter*, *UCG-002*, *Clostridium_sensu_stricto_1*, *Lachnospiraceae_XPB1014_group*, and *Methanobrevibacter*. As shown in Figure 3G, at the genus level, the relative abundance of *NK4A214_group* decreased linearly with increasing dietary NDF intake (*P_linear_* < 0.05). From the LEfSe (Linear discriminant analysis Effect Size) analysis, the magnitude of the impact of the abundance of each component (species) in the treatment group on the differential effect can be determined; a longer bar indicates a more significant difference (Figure 3H). Specifically, the LDA score plot on the right (with an LDA threshold of 4) shows that group A enriched *Firmicutes* at the phylum level and *NK4A214_group* at the genus level, group B enriched *Christensenellaceae* at the family level and *Christensenellales* at the order level, and group E enriched *Lachnospiraceae* at the family level and *Lachnospirales* at the order level.

## 4. Discussion

Constipation is a prevalent issue in gestating sows, predominantly arising from restricted feeding practices and limited movement, which lead to gastrointestinal dysfunction and heightened physiological stress. Gestating sows are extremely prone to constipation due to restricted feeding and housing (limited movement), and in the late gestation stage, as the fetus gradually develops and occupies the intestinal space. Constipation can cause gastrointestinal dysfunction, intestinal microbiota imbalance, increased metabolic burden, intestinal inflammation, and further induce systemic inflammatory responses in sows [1]. Vacuous chewing is a common stereotypic behavior of caged sows. This behavior reflects the psychological stress, maladaptation to the environment, and stress state of sows, and is considered an indicator of poor animal welfare [26,27]. Studies have shown that reducing stereotypic behavior helps to improve the reproductive performance of sows [26,28,29]. The results of this study demonstrate that increasing dietary NDF intake significantly alleviated constipation among gestating gilts, which was consistent with previous studies [30,31]. Specifically, groups receiving higher NDF diets (Groups D and E) exhibited reduced rates of constipation and stereotypic behaviors, underscoring the benefit of dietary fiber in promoting gut health and comfort during gestation. Notably, the prevalence of vacuous chewing—a key stereotypic behavior—decreased significantly in these high NDF intake groups at both day 60 and day 90 of gestation. The reduction in vacuous chewing and alleviation of constipation can be attributed to the properties of dietary fiber. A study by Crome et al. found that beet pulp (predominantly soluble fiber) reduces constipation more effectively due to its strong water-holding capacity, while distillers’ dried grains with solubles (DDGS, mainly insoluble fiber) better mitigates stereotypic behaviors by prolonging sows’ chewing time via its coarser, harder texture [32].

The endocrine and metabolic status of sows are affected by various factors such as dietary composition, gestation stage, and environmental control. Dietary fiber can be fermented by microorganisms in the intestine to produce short-chain fatty acids (SCFAs) such as acetic acid, propionic acid, and butyric acid. These fatty acids can inhibit cholesterol synthesis, thereby reducing plasma cholesterol levels [33]. In this experiment, plasma alkaline phosphatase and cholesterol levels of gilts changed on day 60 of gestation, but this difference disappeared in the subsequent plasma indicators during gestation, which may be related to the physiological metabolism of gilts in the early stage of gestation. Epinephrine and cortisol are indicators reflecting the intensity of stress. Studies have shown that high-fiber diets can reduce animal stress by reducing stereotypic behaviors that cause increases in cortisol and epinephrine [34]. In this experiment, with the increase in dietary NDF intake during gestation, the plasma epinephrine content of sows on the 90th day of gestation decreased linearly, and stereotypic behaviors also decreased. This may be because NDF diets reduce stereotypic behaviors in sows, alleviate sow anxiety, and thereby decrease the secretion of epinephrine in replacement gilts [34,35]. Progesterone is a key hormone for sows to establish and maintain gestation. Studies have shown that high-NDF diets can lead to a decrease in plasma progesterone content of sows throughout the second gestation period [36]. In this experiment, with the increase in dietary NDF intake, the plasma progesterone content of sows decreased linearly on day 90 of gestation, which was consistent with the research results in humans [37]. Fiber has the effect of adsorbing the enterohepatic circulation of steroid hormones, thus promoting the clearance of circulating steroid hormones. A decrease in circulating steroid concentration will reduce its negative feedback on the hypothalamic-pituitary axis, increase the pulse frequency of luteinizing hormone, promote the maturation of oocytes, and improve embryo survival rate [38]. Another study also indicated that different NDF intake can affect the secretion of progesterone in gestating sows, which may be related to the inhibition of cholesterol synthesis by short-chain fatty acids produced by fiber fermentation [39].

During the gestation period of sows, especially in the late gestation stage, due to the rapid development of fetus and mammary glands, the metabolic burden of the mother increases, making them more prone to oxidative stress [40]. Oxidative stress may lead to a decrease in feed intake of sows, hindered fetal development, and even abortion in severe cases [41]. MDA is a product of lipid peroxidation in the body, and its content reflects the degree of oxidative stress [42]. GSH-Px, CAT, and T-SOD are important components of the body’s antioxidant enzyme system and can scavenge accumulated free radicals in the body. Previous studies have found that adding fibers such as inulin, wheat bran, soybean hulls, and beet pulp to the diet can increase the activity of glutathione peroxidase or reduce the content of malondialdehyde [43,44]. This study found that increasing the dietary NDF intake during gestation led to a decrease in plasma MDA content and increases in CAT activity in gilts on day 60 of gestation as well as the total antioxidant capacity (T-AOC) in gilts on day 90 of gestation. The improvement in antioxidant capacity may be related to the higher content of alfalfa hay in high-fiber diets. Studies have shown that alfalfa leaf meal is rich in various nutrients, including vitamins, minerals, and bioactive molecules [45]. These components can increase the activity of antioxidant enzymes in serum, such as SOD and CAT, thereby enhancing the body’s antioxidant capacity and reducing MDA content. However, during the parturition period, when dietary intake of NDF in the gilts diet was increased, the plasma activity of T-SOD was significantly decreased, while the content of MDA was significantly increased. In combination with the characteristic of intense physiological stress imposed on sows during the parturition process, it is speculated that this phenomenon may be due to the excessive intensity of parturition stress, which disrupts the oxidative metabolic homeostasis of sows [46].

Constipation is closely related to intestinal microbiota. The long-term storage of feces in the intestine will further aggravate the imbalance of intestinal microbiota and induce inflammation. In addition, intestinal microbiota plays an important role in the metabolic process of gestating sows, including the absorption and digestion of nutrients, energy acquisition, carbohydrate metabolism, and immune system function [47,48]. In this study, 16S rRNA sequencing technology was used to detect the effect of different dietary NDF intake on the intestinal microbiota of gilts. We employed a subsample size of 6 sows per group for microbiota sequencing, a practice consistent with established literature to ensure reliable detection of microbial community differences [49]. The current results showed that at the phylum level, the top three dominant bacterial phyla were Firmicutes, Bacteroidetes, and Proteobacteria, which was consistent with previous research results [50]. The effect of dietary fiber on intestinal microbiota may depend on the type of fiber, the added fiber level, and individual differences among sows. In this experiment, changing the dietary NDF intake in the diet had no significant effect on the diversity and structure of the intestinal microbiota of sows. Previous studies have found that as age increases, the composition of the microbial community in pigs becomes less dependent on the environment [51]. In this experiment, the pregnant sows were older, and their intestinal microbiota was relatively mature and stable, making it less susceptible to external influences. This also explains why the diversity of fecal microbiota in this study was less affected by the dietary NDF intake. At the phylum level, the relative abundance of Verrucomicrobia increased by higher intake of NDF during gestation. Verrucomicrobia is mainly present in the inner layer of the intestinal mucosa. It is involved in the decomposition of polysaccharides such as mucopolysaccharides and cellulose, thereby providing energy and nutrients for the organism. Verrucomicrobia can utilize mucins in the intestinal mucus layer as the primary carbon source. By degrading mucins, Verrucomicrobia not only supports its own growth but also promotes the renewal and reconstruction of the intestinal mucus layer. Metabolites from mucin degradation stimulate the proliferation of intestinal epithelial cells and enhance the expression of tight junction proteins, thereby strengthening the integrity of the intestinal barrier and promoting gut health [52,53]. Maintaining an appropriate thickness of the intestinal mucus layer can reduce the risk of epithelial cell invasion by pathogenic bacteria, exerting a protective effect [54]. In addition, Verrucomicrobia can also produce short-chain fatty acids (SCFAs), which play an important role in the regulation of intestinal health and the immune system [55,56]. Verrucomicrobia can participate in the decomposition of organic matter and interact with other microorganisms. Verrucomicrobia forms a synergistic interaction with other probiotics through cross-feeding. For instance, its metabolites can promote the growth of Bifidobacterium and Lactobacillus, thereby further optimizing the intestinal microbiota ecology [57]. At the genus level, the relative abundance of *NK4A214* decreased with the increase in dietary NDF intake. *NK4A214*, a bacterial group under the family *Ruminococcaceae*, while certain other groups within *Ruminococcaceae* are important butyrate producers in the gut [58]. Butyrate, a type of short-chain fatty acid (SCFA), has been shown to benefit intestinal development, maintain intestinal health in mammals, and exhibit immune defense functions [59]. The abundance of Verrucomicrobia increased with the elevation of NDF level, while that of *NK4A214* decreased. This variation in their abundance may be attributed to the fact that diets with lower NDF intake contained a higher proportion of barley bran, whereas those with higher NDF intake had a higher intake of alfalfa hay. Different types of fiber exert distinct effects on different intestinal bacterial groups [60,61]. In general, adding NDF to the diet has a certain impact on the intestinal microbiota of sows, but the improvement effect on sow constipation may be more derived from the physical and chemical properties of the fiber itself.

The reproductive performance and overall health of sows are paramount to sustainable pig production, with gestation being a critical period that significantly affects the sow’s well-being and the subsequent litter’s success. Constipation in gestating sows often arises due to restricted feeding practices and limited movement, leading to gastrointestinal dysfunction and increased physiological stress. As such, ensuring the proper nutritional composition of sow diets, particularly through the inclusion of NDF, is essential in fostering optimal reproductive outcomes and health. In this study, the daily net energy intake of sows was calculated based on the National Research Council (NRC, 2012) guidelines, considering the actual body weight of the experimental sows and the expected number of piglets, to ensure that the daily net energy intake level was consistent across all groups. Specifically, the daily net energy intake was 22.0 MJ/d during the 31st to 90th days of gestation, and 26.7 MJ/d during the 91st to 114th days of gestation. Therefore, it was normal that there were no significant differences in backfat thickness and body weight changes among sows in each group during gestation. In this experiment, different dietary NDF intake during gestation had no significant effect on the backfat thickness or body weight of sows, indicating that dietary NDF intake within a certain range do not significantly change the body condition of sows during gestation. However, increasing the dietary NDF intake during gestation can reduce the body weight loss of sows during lactation and improve their body condition. Previous studies have shown that increasing fiber intake during gestation can improve the feed intake of sows in the subsequent lactation period and reduce backfat loss of sows during lactation [62].

## 5. Conclusions

Increasing the NDF content in feed can effectively alleviate constipation in gilts, reduce stereotypic behaviors, and decrease the magnitude of weight loss during the lactation period, without affecting their reproductive performance. Considering the health status and animal stress of sows, the appropriate dietary neutral detergent fiber (NDF) intake levels for sows in the mid-to-late gestation period are 541.81 g/d during days 31~90 of gestation and 657.43 g/d during days 91~114 of gestation, or 588.09 g/d during days 31~90 of gestation and 712.27 g/d during days 91~114 of gestation.

## Figures and Tables

**Table 1:** Daily feed amount and daily intake of neutral detergent fiber per gilt ^a^.

Item	A	B	C	D	E
Daily feed amount					
31~90 d, kg	2.10	2.20	2.29	2.39	2.51
91~114 d, kg	2.55	2.66	2.77	2.90	3.04
Daily intake of neutral detergent fiber					
31~90 d, g	404.88	469.92	505.63	541.81	588.09
91~114 d, g	491.64	568.18	611.62	657.43	712.27
Daily intake of net energy					
31~90 d, MJ	21.98	22.10	22.05	22.01	22.06
91~114 d, MJ	26.70	26.73	26.65	26.70	26.72

^a^ Abbreviation: A (NDF levels = 19.28%); B (NDF levels = 21.36%); C (NDF levels = 22.08%); D (NDF levels = 22.67%); E (NDF levels = 23.43%).

**Table 2:** Composition and nutrient levels of the basal diet (as-fed basis, %) ^a^.

Item	A	B	C	D	E
Ingredients, %					
Corn	49.44	49.32	49.46	49.47	49.79
Soybean meal	5.11	5.17	5.05	4.95	4.86
Rapeseed meal	12.08	9.03	6.25	3.47	0.60
Alfalfa hay	3.63	8.53	13.42	18.30	23.19
Wheat bran	19.00	18.32	17.37	16.52	15.61
Soybean oil	6.67	5.69	4.66	3.67	2.47
L-lysine hydrochloride	0.05	0.06	0.07	0.08	0.10
L-threonine	0.02	0.03	0.04	0.03	0.03
Sodium chloride	0.40	0.40	0.40	0.40	0.40
Limestone powder	0.92	0.79	0.64	0.51	0.36
Dicalcium phosphate	0.46	0.44	0.42	0.40	0.38
Premix ^b^	2.21	2.21	2.21	2.21	2.21
Total	100	100	100	100	100
Nutrient level ^c^					
Net energy, MJ/kg	10.47	10.05	9.62	9.21	8.79
Crude protein, %	15.88	15.32	14.40	13.77	13.33
Crude fiber, %	6.32	7.16	8.09	8.34	8.98
Crude fat, %	8.98	7.71	6.80	6.68	5.73
Starch, %	38.29	38.14	37.21	37.42	36.29
Neutral detergent fiber, %	19.28	21.36	22.08	22.67	23.43
Acid detergent fiber, %	8.67	9.75	10.56	11.19	11.78
SID arginine, %	0.69	0.66	0.62	0.58	0.54
SID histidine, %	0.32	0.30	0.29	0.27	0.25
SID isoleucine, %	0.42	0.40	0.39	0.38	0.37
SID leucine, %	0.94	0.92	0.90	0.88	0.86
SID lysine, %	0.53	0.51	0.49	0.47	0.45
SID methionine, %	0.21	0.20	0.19	0.18	0.17
SID phenylalanine + tyrosine, %	0.83	0.78	0.72	0.67	0.62
SID threonine, %	0.42	0.42	0.41	0.39	0.38
SID tryptophan, %	0.09	0.09	0.08	0.08	0.08
SID valine, %	0.52	0.50	0.48	0.47	0.45
Calcium, %	0.63	0.61	0.58	0.56	0.53
STTD phosphorus, %	0.28	0.27	0.26	0.25	0.24

^a^ Abbreviation: A (NDF levels = 19.28%); B (NDF levels = 21.36%); C (NDF levels = 22.08%); D (NDF levels = 22.67%); E (NDF levels = 23.43%); SID = standard ileal digestible. ^b^ The premix provides the following per 1 kg of diet: vitamin A 10,000 IU, vitamin D 1400 IU, vitamin E 40 mg, vitamin K 3.0 mg, vitamin B 10.50 mg, vitamin B12 0.04 mg, niacin 45 mg, pantothenic acid 20 mg, folic acid 1.2 mg, biotin 0.20 mg, choline chloride 550 mg, copper 80 mg, iron 100 mg, iron 100 mg, zinc 100 mg, manganese 50 mg, iodine (I) 0.3 mg, and selenium 0.25 mg. ^c^ Nutrient Composition and Detection Methods: Crude protein (GB/T 6432-2018) [21], crude fat (GB/T 6433-2006) [22], crude fiber (GB/T 6434-2006) [23], starch (Equl Megazyme), neutral detergent fiber (GB/T 20806-2006) [24], and acid detergent fiber (NY/T 1459-2007) [25] are determined values, while the remaining nutrients are calculated values.

**Table 3:** Effect of dietary different NDF intake during pregnancy on the body weights of gilts ^a^.

Item	A	B	C	D	E	SEM	*p*-Value ^b^		
							ANOVA	Linear	Quadratic
Sow BF thickness during gestation, mm									
Day 30	19.60	19.56	19.78	18.71	19.10	0.33	0.848	0.439	0.875
Day 60	19.56	19.92	19.82	18.64	19.19	0.31	0.685	0.356	0.782
Day 90	19.76	19.91	20.25	19.07	19.27	0.31	0.758	0.407	0.588
Sow BF thickness during lactation, mm									
Farrowing	19.80	19.92	20.32	19.12	19.34	0.34	0.815	0.474	0.624
Weaning	16.33	16.06	16.66	15.79	15.67	0.28	0.796	0.424	0.610
Backfat loss	3.47	3.86	3.65	3.33	3.67	0.15	0.857	0.901	0.879
BW during gestation, kg									
Day 30	198.33	201.06	196.03	196.60	196.93	1.55	0.883	0.529	0.970
Day 60	214.30	219.82	213.29	216.05	216.35	1.55	0.754	0.976	0.930
Farrowing	241.75	248.00	238.24	238.38	247.08	1.87	0.307	0.937	0.342
Lactation									
Feed intake, kg/d	4.95	5.19	5.07	5.34	5.02	0.05	0.152	0.446	0.101
BW at farrowing, kg	221.62	229.33	219.83	218.40	226.35	1.77	0.271	0.905	0.560
BW at Weaning, kg	204.51	208.90	207.95	211.14	212.69	1.81	0.654	0.149	0.920
BW loss, kg	17.11	20.44	11.88	7.26	13.66	1.60	0.101	0.070	0.442

^a^ Abbreviation: A (NDF levels = 19.28%); B (NDF levels = 21.36%); C (NDF levels = 22.08%); D (NDF levels = 22.67%); E (NDF levels = 23.43%); BW = body weight; BF = back fat. ^b^ The *p*-values indicate the effects of dietary NDF intake by one-way ANOVA and linear and quadratic analyses, respectively.

**Table 4:** Effect of Different Dietary Neutral Detergent Fiber intake During Pregnancy on Reproductive Performance of Gilts and Second-Parity Sows ^a^.

Item	A	B	C	D	E	SEM	*p*-Value ^b^		
							ANOVA	Linear	Quadratic
Reproductive performance of gilts at first parity									
Total piglets born, *n*	15.33	14.75	15.00	15.72	16.40	0.34	0.631	0.200	0.305
Live piglets, *n*	11.17	10.83	10.14	10.67	11.07	0.30	0.845	0.863	0.296
Low-birth-weight piglets ^c^, *n*	2.56	2.92	3.14	3.28	2.60	0.29	0.904	0.827	0.382
Stillbirths, *n*	1.17	0.83	1.43	1.28	2.20	0.19	0.254	0.057	0.264
Mummified fetuses, *n*	0.44	0.00	0.07	0.33	0.53	0.07	0.062	0.259	0.008
Litter birth weight, kg	18.24	18.12	17.91	18.01	20.22	0.37	0.241	0.130	0.106
Placental weight, kg	2.88	3.46	2.87	2.83	3.12	0.10	0.341	0.832	0.984
Duration of parturition, min	173.28	180.17	177.14	175.67	160.80	9.29	0.977	0.657	0.601
Estrus rate after weaning, %	50.00	23.50	36.80	50.00	55.00	-	-	-	-
Breeding rate in next parity, %	100.00	92.86	80.00	94.44	93.75	-	-	-	-
Average weight of piglets during lactation									
Day 0, kg	1.44	1.46	1.45	1.44	1.43	0.03	1.000	0.897	0.896
Day 21, kg	5.58	5.74	5.76	5.90	5.81	0.09	0.864	0.343	0.649
Average daily gain of suckling piglets, g/d	197.21	203.98	205.33	212.48	208.53	4.34	0.846	0.314	0.670
Reproductive performance sows at second parity									
Total piglets born	12.82	12.67	13.00	14.22	14.00	0.41	0.665	0.169	0.830
Live piglets	11.54	11.33	11.50	12.33	12.27	0.34	0.838	0.305	0.741
Low-birth-weight piglets, *n*	0.55	0.56	0.88	1.00	0.64	0.14	0.813	0.521	0.435
Stillbirths, *n*	0.27	0.67	0.00	0.44	0.45	0.09	0.274	0.819	0.654
Mummified fetuses, *n*	0.09	0.00	0.25	0.22	0.27	0.06	0.605	0.174	0.992
Malformed piglets, *n*	0.36	0.11	0.38	0.22	0.36	0.10	0.906	0.872	0.664

^a^ Abbreviation: A (NDF levels = 19.28%); B (NDF levels = 21.36%); C (NDF levels = 22.08%); D (NDF levels = 22.67%); E (NDF levels = 23.43%). ^b^ The *p*-values indicate the effects of dietary NDF intake by one-way ANOVA and linear and quadratic analyses, respectively. ^c^ Low birth weight piglets refer to piglets with a birth weight of less than 0.8 kg.

**Table 5:** Effect of dietary different neutral detergent fiber intake during pregnancy on plasma biochemical indices in gilts ^a^.

Item	A	B	C	D	E	SEM	*p*-Value ^b^		
							ANOVA	Linear	Quadratic
Day 60 of gestation									
ALT, U/L	49.94	41.67	43.80	47.23	39.62	2.36	0.676	0.389	0.898
TP, g/L	74.30	75.10	75.60	74.73	73.94	0.63	0.941	0.820	0.423
CREA, umol/L	185.50	197.61	185.21	195.70	188.77	3.48	0.726	0.858	0.619
AST, U/L	34.47	35.24	29.45	34.87	31.85	1.86	0.860	0.687	0.825
ALP, U/L	53.54 ^a,b^	60.90 ^a,b^	55.86 ^a,b^	71.45 ^a^	49.70 ^b^	2.46	0.039	0.853	0.050
ALB, g/L	44.96	45.52	47.37	46.00	46.37	0.66	0.846	0.508	0.539
UREA, mmol/L	5.52	4.80	5.95	5.37	4.85	0.17	0.153	0.498	0.326
GLU, mmol/L	3.35	3.02	2.99	2.52	2.83	0.12	0.270	0.070	0.386
TG, mmol/L	0.30	0.38	0.43	0.41	0.50	0.03	0.181	0.021	0.781
CHO, mmol/L	1.86 ^a,b^	1.85 ^a,b^	1.69 ^b^	1.86 ^a,b^	2.21 ^a^	0.05	0.017	0.034	0.009
HDL, mmol/L	0.81	0.86	0.83	0.92	0.89	0.02	0.394	0.102	0.779
LDL, mmol/L	0.81	0.83	0.83	0.74	0.92	0.03	0.571	0.606	0.389
Day 90 of gestation									
ALT, U/L	38.54	49.37	46.70	39.00	44.45	3.27	0.816	0.952	0.586
TP, g/L	71.17	75.62	71.81	74.68	77.40	0.86	0.107	0.053	0.635
CREA, umol/L	190.02	191.28	202.75	208.64	179.95	4.84	0.377	0.935	0.116
AST, U/L	38.85	77.73	69.71	69.09	47.61	7.05	0.371	0.859	0.064
ALP, U/L	41.68	52.50	47.23	59.10	45.49	2.55	0.230	0.422	0.136
ALB, g/L	49.17	50.86	48.91	50.37	52.55	0.86	0.708	0.330	0.562
UREA, mmol/L	3.64	3.75	4.35	3.81	3.86	0.15	0.653	0.660	0.344
GLU, mmol/L	2.93	3.37	3.41	3.21	3.55	0.10	0.390	0.150	0.614
TG, mmol/L	1.04	0.84	1.08	1.59	1.12	0.12	0.437	0.316	0.799
CHO, mmol/L	1.75	1.84	1.73	1.91	1.99	0.05	0.498	0.155	0.541
HDL, mmol/L	0.84	0.94	0.94	1.05	0.92	0.03	0.283	0.195	0.177
LDL, mmol/L	0.97	0.96	0.87	1.08	1.08	0.04	0.530	0.279	0.417
Farrowing									
ALT, U/L	40.09	44.87	42.67	40.23	40.54	1.19	0.690	0.669	0.380
TP, g/L	67.22	62.57	63.46	65.74	67.85	1.03	0.433	0.550	0.098
CREA, umol/L	206.08	225.62	206.39	207.43	188.68	6.88	0.605	0.297	0.348
AST, U/L	38.66	47.32	43.23	45.77	43.71	2.48	0.865	0.647	0.504
ALP, U/L	51.42	85.38	61.00	49.96	50.94	5.53	0.206	0.342	0.248
ALB, g/L	49.22	46.45	46.18	46.79	54.01	1.04	0.079	0.154	0.015
UREA, mmol/L	4.65	5.54	4.40	4.44	4.96	0.23	0.511	0.770	0.817
GLU, mmol/L	4.18	6.01	4.93	4.45	5.07	0.24	0.153	0.903	0.359
TG, mmol/L	1.07	0.90	1.04	0.70	0.66	0.10	0.593	0.163	0.790
CHO, mmol/L	1.50	1.27	1.48	1.40	1.47	0.05	0.673	0.870	0.509
HDL, mmol/L	0.67	0.58	0.59	0.59	0.65	0.02	0.616	0.828	0.133
LDL, mmol/L	0.79	0.68	0.83	0.84	0.72	0.04	0.656	0.905	0.654

^a^ Abbreviation: A (NDF levels = 19.28%); B (NDF levels = 21.36%); C (NDF levels = 22.08%); D (NDF levels = 22.67%); E (NDF levels = 23.43%); alkaline phosphatase (ALP); total protein (TP); creatinine (CREA); aspartate aminotransferase (AST); alkaline phosphatase (ALP); albumin (ALB); urea (UREA); glucose (GLU); triglycerides (TG); cholesterol (CHO); high-density lipoprotein cholesterol (HDL); low-density lipoprotein cholesterol (LDL). ^b^ The *p*-values indicate the effects of dietary NDF intake by one-way ANOVA and linear and quadratic analyses, respectively. ^a,b^ Values within a row with different superscripts differ significantly at *p* < 0.05.

**Table 6:** Effect of dietary different neutral detergent fiber intake during pregnancy on plasma antioxidant capacity in gilts ^a^.

Item	A	B	C	D	E	SEM	*p*-Value ^b^		
							ANOVA	Linear	Quadratic
Day 60 of gestation									
T-AOC, U/mL	3.28 ^a,b^	4.44 ^a^	3.33 ^a,b^	3.70 ^a^	2.26 ^b^	0.19	0.002	0.015	0.007
TSOD, U/mL	167.48	161.14	156.70	188.14	205.73	7.16	0.151	0.040	0.152
MDA, nmol/mL	5.90 ^a,b^	6.84 ^a^	3.15 ^b^	4.22 ^a,b^	3.37 ^b^	0.41	0.006	0.004	0.688
GSH-Px, U/mL	1075.56 ^a,b^	1446.46 ^a^	1100.61 ^a,b^	1014.14 ^b^	1049.70 ^b^	47.92	0.017	0.107	0.241
CAT, U/mL	20.29 ^a,b^	27.18 ^a,b^	33.72 ^a,b^	15.39 ^b^	40.69 ^a^	2.82	0.022	0.104	0.563
Day 90 of gestation									
T-AOC, U/mL	1.37 ^b^	2.24 ^a,b^	2.63 ^a,b^	3.24 ^a,b^	3.86 ^a^	0.28	0.046	0.002	0.903
TSOD, U/mL	117.66	148.20	156.48	178.45	143.66	8.57	0.267	0.176	0.107
MDA, nmol/mL	1.53	2.33	2.42	0.73	1.93	0.25	0.191	0.640	0.629
GSH-Px, U/mL	691.20	760.53	752.00	797.87	779.73	21.30	0.605	0.176	0.514
CAT, U/mL	21.68 ^b^	40.24 ^a^	34.29 ^a,b^	38.17 ^a^	33.72 ^a,b^	1.94	0.014	0.069	0.014
Farrowing									
T-AOC, U/mL	2.33	2.23	1.03	1.46	1.28	0.17	0.055	0.015	0.036
TSOD, U/mL	286.83 ^a^	230.98 ^b^	254.18 ^a,b^	258.82 ^a,b^	233.83 ^b^	5.45	0.001	0.025	0.039
MDA, nmol/mL	1.25 ^b^	3.65 ^a^	2.54 ^a^	3.80 ^a^	2.96 ^a^	0.25	0.003	0.040	0.013
GSH-Px, U/mL	885.10	814.37	858.40	768.15	820.23	14.52	0.119	0.094	0.123
CAT, U/mL	33.31 ^b^	65.27 ^a^	73.51 ^a^	51.19 ^a,b^	27.50 ^b^	4.02	<0.001	0.329	<0.001

^a^ Abbreviation: A (NDF levels = 19.28%); B (NDF levels = 21.36%); C (NDF levels = 22.08%); D (NDF levels = 22.67%); E (NDF levels = 23.43%); total antioxidant capacity (T-AOC); total superoxide dismutase (T-SOD); malondialdehyde (MDA); glutathione peroxidase (GSH-Px); catalase (CAT). ^b^ The *p*-values indicate the effects of dietary NDF intake by one-way ANOVA and linear and quadratic analyses, respectively. ^a,b^ Values within a row with different superscripts differ significantly at *p* < 0.05.

**Table 7:** Effect of dietary different neutral detergent fiber intake at day 90 of gestation on plasma hormone, IgA, IgM and IgG indices in gestating gilts ^a^.

Item	A	B	C	D	E	SEM	*p*-Value ^b^		
							ANOVA	Linear	Quadratic
P4, pmol/L	1535.00 ^a^	1485.00 ^a,b^	1460.00 ^a,b^	1229.17 ^a,b^	1153.33 ^b^	46.11	0.016	0.001	0.441
E2, pmol/L	130.35	130.14	123.47	135.21	115.76	3.39	0.433	0.325	0.486
INS, mIU/L	60.21	54.98	57.89	58.31	53.66	1.06	0.298	0.196	0.879
EPI, ng/L	133.51 ^a^	126.72 ^a,b^	115.31 ^a,b^	120.79 ^a,b^	103.31 ^b^	3.33	0.035	0.004	0.857
GHR, ng/L	925.13	843.75	953.13	1100.00	1021.88	41.34	0.371	0.138	0.885
COR, μg/L	127.44	121.25	135.24	127.86	134.82	2.74	0.484	0.285	0.833
IgA,ug/mL	26.34	23.95	25.85	24.61	21.30	0.56	0.024	0.014	0.025
IgG,ug/mL	286.88	255.00	247.71	228.13	212.50	8.04	0.029	0.001	0.004
IgM.ug/mL	29.51	25.44	24.77	25.91	18.98	0.92	0.002	0.001	0.003

^a^ Abbreviation: A (NDF levels = 19.28%); B (NDF levels = 21.36%); C (NDF levels = 22.08%); D (NDF levels = 22.67%); E (NDF levels = 23.43%); progesterone (P4); estradiol (E2); insulin (INS); epinephrine (EPI); groth hormone-releasing hormone (GHR); cortisol (COR); immunoglobulin A (IgA); immunoglobulin G (IgG); immunoglobulin M (IgM). ^b^ The *p*-values indicate the effects of dietary NDF intake by one-way ANOVA and linear and quadratic analyses, respectively. ^a,b^ Values within a row with different superscripts differ significantly at *p* < 0.05.

## Data Availability

All the datasets used and analyzed during the current study are included in the manuscript.

## Abbreviations

The following abbreviations are used in this manuscript:

NRC	The National Research Council
NE	net energy
